# Pretreatment of nucleus pulposus mesenchymal stem cells with appropriate concentration of H_2_O_2_ enhances their ability to treat intervertebral disc degeneration

**DOI:** 10.1186/s13287-022-03031-7

**Published:** 2022-07-26

**Authors:** Yu-yao Zhang, Zhi-lei Hu, Yu-han Qi, Hai-yin Li, Xian Chang, Xiao-xin Gao, Chen-hao Liu, Yue-yang Li, Jin-hui Lou, Yu Zhai, Chang-qing Li

**Affiliations:** 1 Department of Orthopedics, Xinqiao Hospital, Army Military Medical University, Chongqing, 400037 China; 2 Institute of Basic Theory of Traditional Chinese Medicine, China Academy of Chinese Medical Science, Beijing, 100000 China

**Keywords:** NPMSCs, H_2_O_2_, Pretreatment, Transplantation, IVDD

## Abstract

**Background:**

Nucleus pulposus mesenchymal stem cells (NPMSCs) transplantation is a promising treatment for intervertebral disc degeneration (IVDD). However, the transplanted NPMSCs exhibited weak cell proliferation, high cell apoptosis, and a low ability to resist the harsh microenvironment of the degenerated intervertebral disc. There is an urgent need to explore feasible methods to enhance the therapeutic efficacy of NPMSCs transplantation.

**Objective:**

To identify the optimal concentration for NPMSCs pretreatment with hydrogen peroxide (H_2_O_2_) and explore the therapeutic efficacy of NPMSCs transplantation using H_2_O_2_ pretreatment in IVDD.

**Methods:**

Rat NPMSCs were pretreated with different concentrations (range from 25 to 300 μM) of H_2_O_2_. The proliferation, reactive oxygen species (ROS) level, and apoptosis of NPMSCs were detected by cell counting kit-8 (CCK-8) assay, 5-ethynyl-2′-deoxyuridine (EdU) staining, and flow cytometry in vitro. The underlying signalling pathways were explored utilizing Western blotting. A rat needle puncture-stimulated IVDD model was established. X-ray, histological staining, and a multimode small animal live imaging system were used to evaluate the therapeutic effect of H_2_O_2_-pretreated NPMSCs in vivo.

**Results:**

NPMSCs pretreated with 75 μM H_2_O_2_ demonstrated the strongest elevated cell proliferation by inhibiting the Hippo pathway (*P* < 0.01). Meanwhile, 75 μM H_2_O_2_-pretreated NPMSCs exhibited significantly enhanced antioxidative stress ability (*P* < 0.01), which is related to downregulated Brd4 and Keap1 and upregulated Nrf2. NPMSCs pretreated with 75 μM H_2_O_2_ also exhibited distinctly decreased apoptosis (*P* < 0.01). In vivo experiments verified that 75 μM H_2_O_2_-pretreated NPMSCs-transplanted rats exhibited an enhanced disc height index (DHI% = 90.00 ± 4.55, *P* < 0.01) and better histological morphology (histological score = 13.5 ± 0.5, *P* < 0.01), which means 75 μM H_2_O_2_-pretreated NPMSCs can better adapt to the environment of degenerative intervertebral discs and promote the repair of IVDD.

**Conclusions:**

Pretreatment with 75 μM H_2_O_2_ was the optimal concentration to improve the proliferation, antioxidative stress, and antiapoptotic ability of transplanted NPMSCs, which is expected to provide a new feasible method to improve the stem cell therapy efficacy of IVDD.

**Supplementary Information:**

The online version contains supplementary material available at 10.1186/s13287-022-03031-7.

## Introduction

IVDD is an important cause of low back pain (LBP) and lower extremity dysfunction, which impose a heavy economic burden on society [1, 2]. The intervertebral disc is an important connection structure between the vertebral bodies that consists of the nucleus pulposus, the annulus fibrosus, and the upper and lower cartilage endplates, which can maintain the amplitude of spinal motion and withstand mechanical pressure [3]. When the intervertebral disc undergoes gradual degeneration, the number of nucleus pulposus cells and the anabolic processes decrease, resulting in a series of clinical symptoms, such as backache, numbness, and lower limb pain [4]. Although surgery is a commonly chosen method for palliating symptoms, surgery fails to restore the biological and mechanical functions of the intervertebral disc and may even alter the biomechanical function of the spine, which may lead to further degeneration of the adjacent intervertebral disc [5, 6]. Therefore, it is necessary to explore new feasible strategies to treat IVDD.

Stem cell transplantation has become a promising therapeutic method utilized to treat IVDD experimentally and clinically in recent years [7, 8]. The intervertebral disc is recognized as an immune-exempt area, and this feature makes it possible to use exogenous stem cells to treat IVDD [9, 10]. Extensive studies have shown that a variety of stem cells can directly differentiate into nucleus pulposus cells and facilitate the function of degenerative nucleus pulposus cells in a paracrine manner when transplanted into the degenerative intervertebral disc [11–14]. Recent studies have identified NPMSCs with mesenchymal stem cell characteristics in the nucleus pulposus tissue, which possess a stronger ability to differentiate into nucleus pulposus cells and withstand the microenvironment of the intervertebral disc compared with commonly used stem cells such as bone marrow mesenchymal stem cells (BMMSCs) and adipose mesenchymal stem cells (ADMSCs) [15–17]. However, the low viability of transplanted stem cells at the transplant site has become a bottleneck for stem cell-based IVDD therapy. The intervertebral disc is the largest tissue without blood supply in the whole body, and the degenerated intervertebral disc exhibits a harsh microenvironment with low nutrition, low pH, high mechanical pressure, and high osmotic pressure, coupled with distinct oxidative stress and local inflammation. Therefore, the transplanted cells will suffer cell cycle arrest and considerable cell death, which immensely undermine the effect of stem cell transplantation and even lead to transplantation failure [18–20].

Numerous methods have been explored to improve the therapeutic effect of transplanted stem cells in IVDD treatment, including conjunction with biological scaffolds, hydrogel materials, genetic modification, and cellular pretreatment, and pretreatment of stem cells with the mimetic harsh environment (e.g., hypoxia, acid, mechanical intervention) can improve their postengraftment survival and differentiation characteristics [21–23]. Among these pretreatments, pretreatment of transplanted cells with H_2_O_2_ is convenient to obtain and simple to perform. Previous studies have shown that pretreatment with an appropriate concentration of H_2_O_2_ can significantly improve the proliferation, paracrine manner, antiapoptotic ability, and therapeutic effect of MSCs in liver fibrosis, skin wounds, and myocardial infarction [24–26]. However, a high concentration of H_2_O_2_ is cytotoxic and will cause an increase in ROS, which may lead to irreversible oxidative damage to the cells [27, 28].

Based on the above studies, we hypothesized that NPMSCs pretreated with an appropriate concentration of H_2_O_2_ may have better biological functions, such as proliferation and antiapoptosis, and may be suitable for stem cell transplantation-based intervertebral disc repair. Therefore, this study aims to verify the optimal concentration of H_2_O_2_ pretreatment and explore whether pretreated NPMSCs possess presumptive therapeutic effects in treating intervertebral disc degeneration.

## Materials and methods

### Ethical statement

Animal experiments were performed on 8-week-old male Sprague Dawley (SD) rats (*n* = 72) provided by the Animal Center of Xinqiao Hospital. NPMSCs were extracted from 30 rats, and 42 rats were used for the cell transplantation experiments. All experiments were approved by the Ethics Committee of the Army Medical University.

### Experimental design and treatment protocol

In vitro: After isolating NPMSCs, we identified the trilineage differentiation capacity (*n* = 3) and detected the characteristic cell surface molecules of mesenchymal stem cells (*n* = 3). The cells were treated with different concentrations of H_2_O_2_ (ranging from 25 to 300 μM), and then cell counting kit-8 (CCK-8) assay experiments (*n* = 3), EdU experiments (*n* = 3), and Western blotting (*n* = 3) were used to explore the optimal concentration of H_2_O_2_ and the possible mechanism to promote the proliferation of NPMSCs. Next, NPMSCs pretreated with the optimal concentration of H_2_O_2_ were exposed to a sublethal dose of H_2_O_2_. Then, intracellular reactive oxygen species (ROS) were detected by a dichlorodifluorofluorescein diacetate (DCFH-DA) probe (*n* = 3), mitochondrial membrane potential (MMP) was detected by a CMXRos probe (*n* = 3), the percentage of apoptosis was detected by an Annexin V-PE/7-AAD probe (*n* = 3), and Western blotting (*n* = 3) was used to explore the possible mechanism. A schematic depiction of the treatment timelines is presented in Additional file 1: Fig. S1.

In vivo: Rats with intervertebral disc degeneration were randomly divided into the following groups: (1) untreated group (*n* = 6); (2) phosphate-buffered saline (PBS) injection group (*n* = 6); (3) NPMSCs group (*n* = 6); (4) 50 μM H_2_O_2_-pretreated NPMSCs group (*n* = 6); (5) 75 μM H_2_O_2_-pretreated NPMSCs group (*n* = 6); and (6) 100 μM H_2_O_2_-pretreated NPMSCs group (*n* = 6). The NPMSCs were labelled with green fluorescent protein (GFP), and X-rays were taken before treatment and on the 30th and 60th days after treatment, and the change in DHI% was calculated. The fluorescence intensity (*n* = 3) and histological changes in the intervertebral disc were detected on the 60th day after treatment. A schematic depiction of the timelines is presented in Fig. 6a.

### Cell preparation

Rats were killed after being properly anaesthetized with 2% pentobarbital (50 mg/kg), and then the caudal vertebrae were isolated after removing the skin, ligaments, and muscle tissues under aseptic conditions. The nucleus pulposus tissue was separated from the caudal intervertebral disc, and then we mixed the nucleus pulposus tissue of every 10 rats and digested it with 0.2% type II collagenase (C2-BIOC, Sigma-Aldrich, USA) for approximately 2 h at 37 °C, followed by centrifugation at 400×g for 5 min at room temperature. The supernatant was discarded and the sediment was suspended and cultured in a standard mesenchymal stem cell growth medium, consisting of Dulbecco modified Eagle medium low glucose (SH30021, HyClone, Logan, USA), 10% foetal calf serum (S711-001S, LONSA, Canelones, UY), and 1% penicillin/streptomycin (C0222, Beyotime, Shanghai, China). NPMSCs were placed in a humidified chamber in a 5% CO_2_–air mixture at 37 °C. The medium was changed every 3–4 days. Cells were subcultured when they reached a confluence of 80–90%. NPMSCs from the third generation were used throughout the experiment.

### Detection of cell surface markers

NPMSCs were digested with 0.25% trypsin (SH30042, HyClone) and suspended in PBS at 5 × 10^5^ cells/ml. Furthermore, 100 µl of cell suspension solution was aliquoted per tube and incubated, respectively, with a solution of fluorescein isothiocyanate (FITC)-conjugated CD34, CD45, CD73, CD90, and CD105 (all from Bioworld, MN, USA) at room temperature for 30 min. An isotype control was used in each case. After incubation, the cells were washed three times with PBS and then centrifuged at 4 °C at 250×*g* for 5 min. The supernatant was discarded, and the cells were resuspended in 500 µl of PBS prior to flow cytometry analyses (Gallios, Beckman, Germany).

### Detection of multilineage differentiation ability

The multilineage differentiation ability of NPMSCs was detected using an osteogenic differentiation induction kit (RASMX-90021, Cyagen Biosciences, Suzhou, China), a chondrogenic differentiation induction kit (RASMX-90041/90042, Cyagen Biosciences), and an adipogenic induction differentiation kit (RASMX-90031, Cyagen Biosciences). After 21 days of induction, NPMSCs were fixed with 4% paraformaldehyde (P0099, Beyotime) for 30 min. Then, Alizarin Red staining, Oil Red O staining, and Alcian Blue staining were performed according to the instructions.

### Cell counting kit-8 assay

The cell viability of the NPMSCs was detected using cell counting kit-8 (C0038, Beyotime). NPMSCs were seeded into 96-well plates at a density of 5 × 10^3^ cells. NPMSCs were able to grow adherent cells in the plates after one night of culture. Then, H_2_O_2_ (323381, Sigma-Aldrich) was added to the medium for 12 h at concentrations of 25, 50, 75, 100, 150, 200, and 300 μM. The medium was replaced with fresh medium. Then, 10 µl CCK‐8 reagent was added to each well and incubated at 37 °C for 2 h. A spectrophotometer (M2, MOLECULAR DEVICE, USA) was used to detect the optical density (OD) values at a wavelength of 450 nm. Each trial was repeated three times.

### EdU staining assay

Cell proliferation activity was detected using an EdU-555 cell proliferation detection kit (C0075S, Beyotime). The cells were inoculated into 6-well plates at a density of 2 × 10^5^ cells per well. The cells were then treated with H_2_O_2_ at concentrations of 25, 50, 75, 100, 150, 200, and 300 μM for 12 h. Meanwhile, we designed four treatment methods for further exploration: untreated group, 75 μM H_2_O_2_ treatment for 12 h, 300 μM H_2_O_2_ treatment for 12 h, 75 μM H_2_O_2_ treatment for 12 h and then 300 μM H_2_O_2_ treatment for 12 h. Two millilitres of 10 μM EdU reagent was added to each well and incubated for 2 h. Cells were fixed at room temperature for 15 min with 2 ml 4% paraformaldehyde. Two millilitres of immunostaining strong permeability solution (P0097, Beyotime) was added to each well and incubated for 15 min. Two millilitres of Click Additive Solution was added to each well and incubated for 30 min at room temperature (shielded from light). The nuclei were stained with Hoechst 33,358 solution (C0021, Solarbio, Beijing, China). Immunofluorescent staining images were captured by a fluorescence microscope (IX73, Olympus, Tokyo, Japan).

### Western blotting

Protein was extracted from NPMSCs by a radioimmunoprecipitation assay (RIPA) solution (P0013B, Beyotime) containing 2% phenylmethanesulfonyl fluoride (PMSF) (ST507, Beyotime) and 2% phosphatase inhibitor (P1082, Beyotime). The protein was then centrifuged at 4 °C and 12,000×*g* for 5 min to collect the supernatant. Protein concentration was detected by bicinchoninic acid (BCA) protein concentration assay kit (PC0020, Solarbio). Then, protein loading buffer (P0015L, Beyotime) was added to each sample and heated in a boiling water bath for 5 min. The protein was separated in a 12% sodium salt-polyacrylamide gel, and then the protein was transferred to polyvinylidene fluoride (PVDF) membranes (ISEQ00010, Merck Millipore, Darmstadt, Germany). The membranes were soaked with 5% skim milk powder (P0216, Beyotime) for 1 h at room temperature and incubated with corresponding antibodies at 4 °C overnight. After washing with tris-buffered saline (TBS) with Tween-20 (TBST), the membranes were immersed in the corresponding horseradish peroxidase-labelled secondary antibody (diluted concentration 1:500) and incubated at room temperature for 1 h. An electrochemiluminescence (ECL) kit (1705060, BIO-RAD, CA, USA) and a gel imaging system (ChemiDoc, Bio-Rad) were utilized to visualize the protein content. Protein bands were analysed using ImageJ software (National Institute of Health, USA). All results were quantified and normalized to β-actin. The antibodies used were as follows: cyclin D1 (ab134175), P16 (ab51243), β-actin (ab8227), cytochrome C (ab133504), Brd4 (ab75898), Keap1 (ab119403), and Nrf2 (ab92946) from Abcam (Cambridge, UK); cleaved caspase-3 (9664T), p-Lats1 (9157), p-Mst1 (49332), p-YAP (13008T), and YAP (14074T) from CST (Shanghai, China). Bcl-2 (26593-1-AP) and Bax (60267-1-Ig) were purchased from Proteintech (Chicago, USA).

### Detection of ROS

ROS levels in cells were detected using an ROS detection kit (S0033M, Beyotime). Two millilitres of 10 μM DCFH-DA (diluted in serum-free medium) was added to each well, and the cells were incubated at 37 °C for 20 min (shielded from light). The cells were then washed with serum-free medium three times to remove the unloaded probes. Then, the cells were collected and the production of ROS was detected by flow cytometry.

### Detection of MMP

MitoTracker Red CMXRos (C1049B, Beyotime) was used to detect MMP. Briefly, NPMSCs were inoculated into 6-well plates at a density of 2 × 10^5^ cells per well and treated with H_2_O_2_ at concentrations of 75 and 300 μM for 24 h, with or without 75 μM H_2_O_2_ for 12 h prior to 300 μM H_2_O_2_ treatment. Then, 1 ml of MitoTracker Red CMXRos working solution was added to each well and incubated at 37 °C for 30 min (shielded from light). The nuclei were stained with Hoechst 33,358 staining solution. The staining was observed under a laser confocal microscope (LSM880, ZEISS, Germany).

### Detection of apoptosis rate

The rate of apoptosis was detected using the Annexin VPE/7-AAD apoptosis detection kit (BD Pharmingen, Franklin Lakes, NJ, USA). NPMSCs were collected and resuspended in binding buffer at a concentration of 5 × 10^6^ cells/ml. Then, 100 µl of cell suspension was transferred to the test tube, and 5 µl of Annexin VPE and 5 µl of 7-AAD were added. After incubating at room temperature for 15 min (shielded from light), 400 µl binding buffer was added to each sample and the apoptosis rate was detected by flow cytometry.

### Labelling NPMSCs with green fluorescent protein (GFP)

Lentiviral vectors expressing GFP (GV493, GeneChem, Shanghai, China) were transfected into NPMSCs according to the manufacturer’s instructions. The multiplicity of infection (MOI) was set at 20, and the transfection time was set at 18 h. Puromycin (5 μg/ml, ST551, Beyotime) was used to select stably transfected NPMSCs.

### Establishment of IVDD model in rats

Forty-two healthy male SD rats aged 8 weeks were randomly selected for animal experiments. Rats were fasted for 12 h before surgery, and water was forbidden for 4 h before surgery. Then, the rats were anaesthetized by intraperitoneal injection of 2% pentobarbital (50 mg/kg). The tail skin of rats was sterilized three times with iodophor, and percutaneous intervertebral disc puncture was performed with a no. 20 needle at the coccyx 6–7 (CO6-7) level [29]. The needle was vertically inserted at a depth of 5 mm and rotated 360° for 30 s before exiting. The surgical areas were then sterilized again with iodophor. Rats were provided with food and water 12 h after the operation to prevent intestinal paralysis.

### NPMSCs transplantation in IVDD rats

Thirty days after the operation, X-ray was used to verify whether IVDD was successfully created. After excluding 3 rats that died accidentally and 3 rats that failed to induce IVDD, the remaining 36 rats with obvious IVDD were randomly divided into 6 groups with different treatments at CO6-7: Untreated group were treated without any treatment, PBS injection group were injected with 2 µl PBS, NPMSCs group were injected with labelled NPMSCs (5 × 10^4^ cells, suspended in PBS), 50 μM H_2_O_2_ pretreated NPMSCs group were injected with labelled NPMSCs (5 × 10^4^cells, suspended in PBS) after 50 μM H_2_O_2_ pretreatment for 12 h, 75 μM H_2_O_2_ pretreated NPMSCs group were injected with labelled NPMSCs (5 × 10^4^ cells, suspended in PBS) after 75 μM H_2_O_2_ pretreatment for 12 h, and 100 μM H_2_O_2_-pretreated NPMSCs group were injected with labelled NPMSCs (5 × 10^4^ cells, suspended in PBS) after 100 μM H_2_O_2_ pretreatment for 12 h. PBS and cells were injected through a microsyringe (25 µl, Gaoge, Shanghai, China).

### Disc height measurement

A multimode small animal live imaging system (Xtreme ll, Bruker, Belgium) was used to obtain X-ray images of rats. Rats were anaesthetized by intraperitoneal injection of 2% pentobarbital (50 mg/kg). Then, the rats were fixed on the tray and put into the apparatus. The parameters were as follows: Modality (X-Ray), Background (None), Illumination (X-Ray), Filters (X-Ray 0.4 mm, KVP 45, Excitation 0, Emission 0), Exposure time (1.2 s), FOV (15CM), fStop (2), Bin (1 × 1), Focal Plane (0 mm), and Focal Zone (X-Ray). Finally, ImageJ was used to measure the three longitudinal lines of the intervertebral disc and the two adjacent upper and lower vertebral bodies, and then the DHI% was calculated according to the method recorded in previous research [9] to evaluate the degree of intervertebral disc degeneration.

### Histological analyses of the caudal intervertebral disc

On the 60th day after treatment, the caudal vertebrae of the rats were collected. The samples were fixed in 4% paraformaldehyde solution for 48 h. Then, rapid bone tissue decalcification solution (BB-23619, BestBio, Shanghai, China) was used for 2 days of decalcification. Then, the samples were dehydrated with ethanol solutions of different concentrations (75%, 85%, 95%, and 100%). Paraffin sections (6 um) were prepared and stained with Haematoxylin–Eosin (HE) Staining Kit (G1120, Solarbio) and a Modified Safranine O-Fast Green FCF Stain Kit (G1371, Solarbio) to evaluate the degree of IVDD in each group. Three observers blindly observed the shape and structure of the intervertebral disc and the type and number of cells in the intervertebral disc and scored them using the grading scale (Additional file 2: Table S1) reported in previous research [30].

### Fluorescence imaging of rats in vivo

A multimode small animal live imaging system (Xtreme ll, Bruker) was used to obtain fluorescence images of rats. Rats were anaesthetized by intraperitoneal injection of 2% pentobarbital (50 mg/kg). Then, we fixed the rats on the tray and placed them into the apparatus. The parameters were as follows: Modality (Fluorescence), Background (None), Illumination (Multi-wavelength), Filters (X-Ray 0 mm, KVP 45, Excitation 470, Emission 535), Exposure time (2 s), FOV (15CM), fStop (2), Bin (2 × 2), Focal Plane (0 mm), and Focal Zone (Tray).

### Statistical analysis

All experiments were repeated three times with at least triplicate samples. The results are presented as the mean ± standard deviation (SD). Statistical analysis was performed using SPSS 23.0 (SPSS Inc. IL, New York, NY, USA). Multiple comparisons were made using one-way or two-way analysis of variance (ANOVA) followed by Bonferroni’s post hoc test. The DHI% data were analysed by two-way repeated-measures ANOVA followed by Bonferroni’s post hoc test. A value of *P* < 0.05 was considered statistically significant.

## Results

### Identification of NPMSCs

**Fig. 1:**
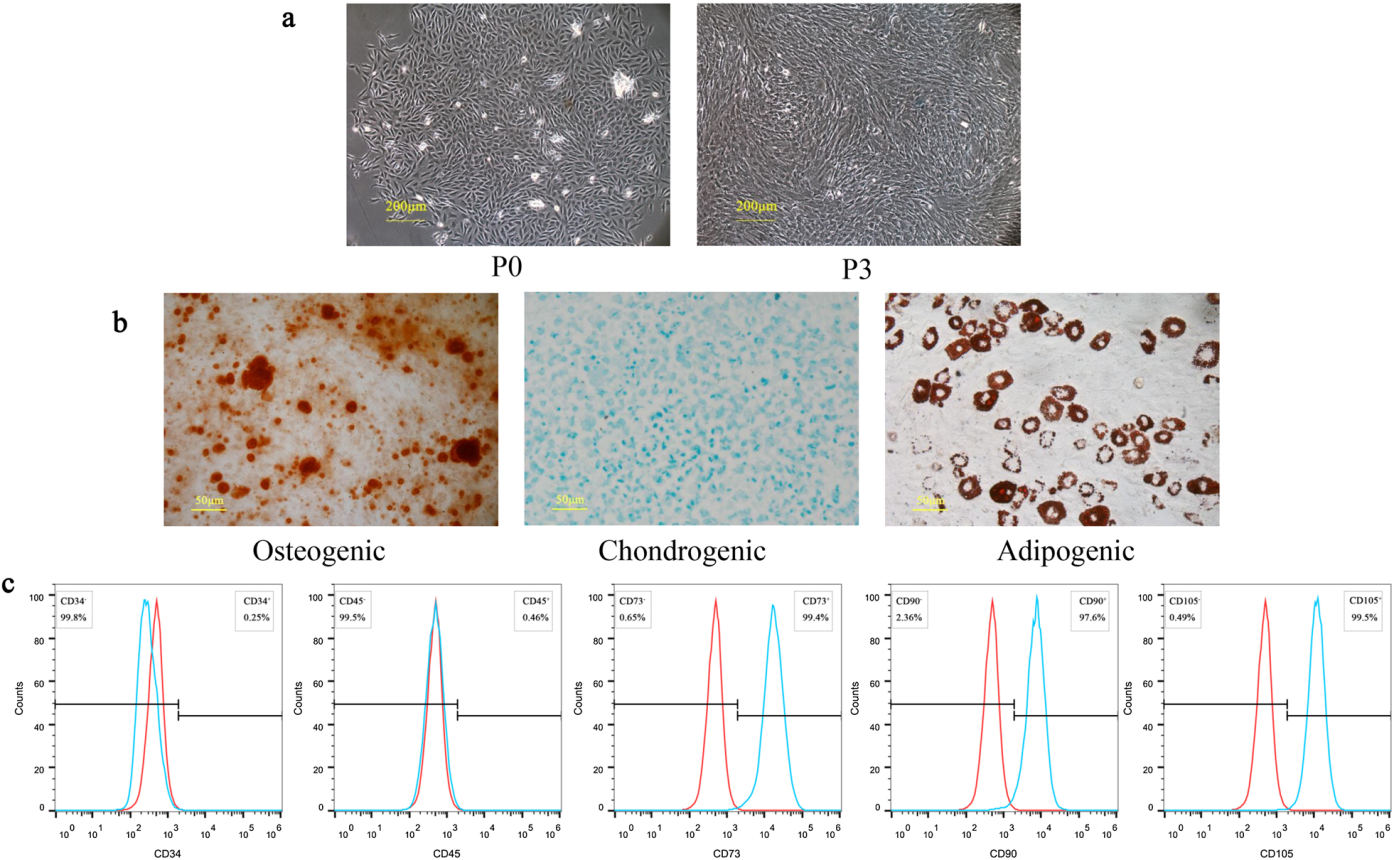
Identification of NPMSCs. **a** Representative image illustrating the morphology of NPMSCs. Scale bar = 200 μm. **b** Alizarin Red staining, Alcian Blue staining, and Oil Red O staining results of NPMSCs at 21 days post-induction. Scale bar = 50 μm. **c** Flow cytometry examining the expression of CD34, CD45, CD73, CD90, and CD105 on NPMSCs

NPMSCs were isolated from the rat caudal intervertebral disc. After 5 days of culture, the primary cells exhibited a short spindle shape. The third generation of NPMSCs showed accelerated and spiral cell growth (Fig. 1a).

The multilineage differentiation ability of NPMSCs was assessed in the third generation of NPMSCs. After induction for 21 days, Alizarin Red staining showed the formation of mineralized nodules after osteogenic differentiation induction. Alcian Blue staining showed distinct sulphurated proteoglycan staining after chondrogenic differentiation induction. Oil Red O staining showed lipid vacuole formation after adipogenic differentiation induction (Fig. 1b). Flow cytometry results showed that the expression of CD73, CD90, and CD105 was positive (more than 95%) and CD34and CD45 were negative (less than 5%) in NPMSCs (Fig. 1c).

### H_2_O_2_ (75 μM) promoted the proliferation of NPMSCs by inhibiting the Hippo signalling pathway

**Fig. 2:**
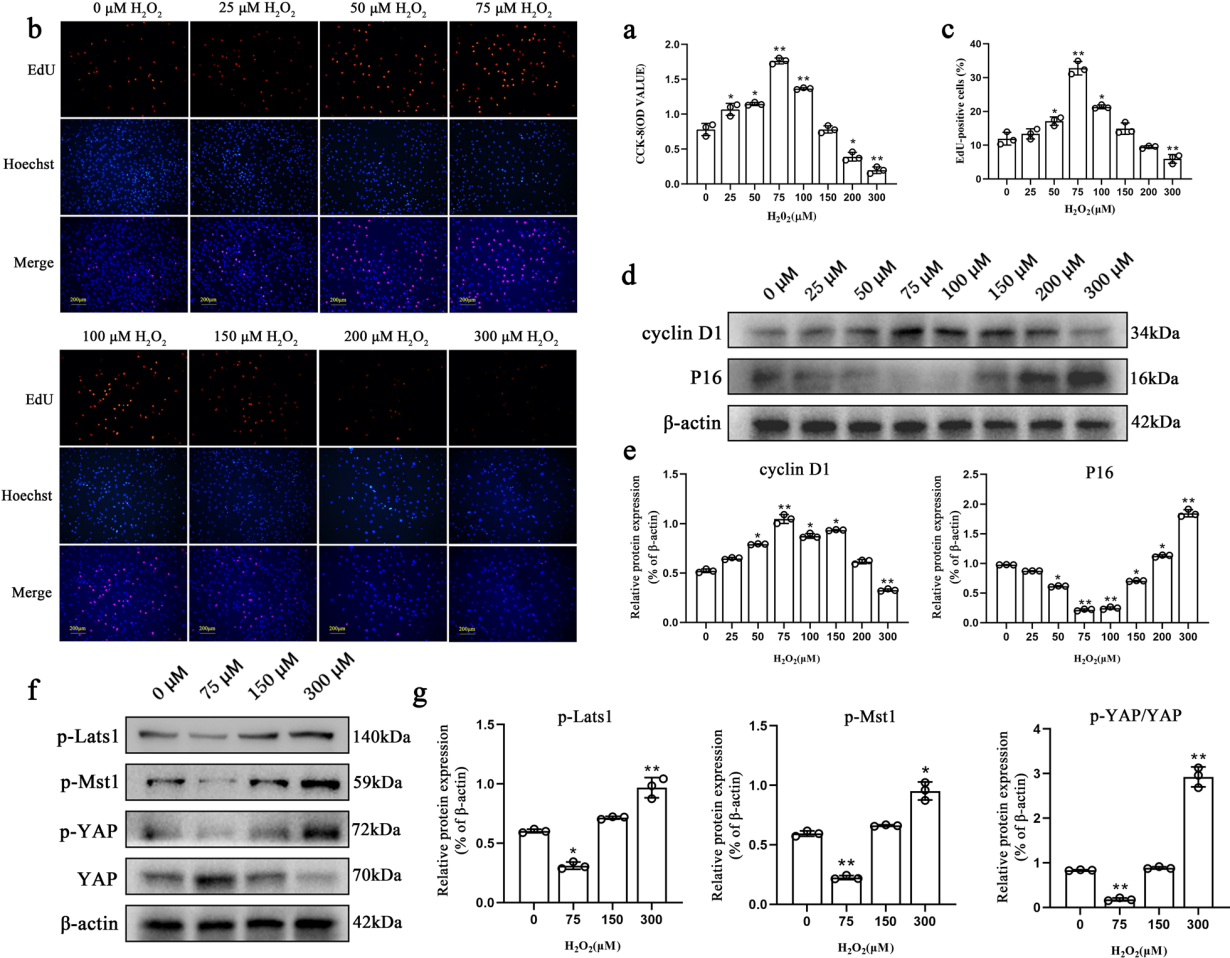
Effects of different H_2_O_2_ concentrations on NPMSCs proliferation. **a** The effect of H_2_O_2_ on NPMSC proliferation detected by the CCK-8 assay. (*n* = 3, *P < 0.05, **P < 0.01, one-way ANOVA followed by Bonferroni’s post hoc test). **b** and **c** Detection of proliferated NPMSCs (red) under different H_2_O_2_ concentrations by the EdU-555 proliferation assay kit and quantification. Nuclei were counterstained with Hoechst 33358 (blue) (*n* = 3, *P < 0.05, **P < 0.01, one-way ANOVA followed by Bonferroni’s post hoc test). Scale bar = 200 μm. **d** and **e** Western blotting analysis of cyclin D1 and P16 expression in NPMSCs exposed to different concentrations of H_2_O_2_ for 12 h (*n* = 3, *P < 0.05, **P < 0.01, one-way ANOVA followed by Bonferroni’s post hoc test). **f** and **g** Western blotting analysis of p-Lats1, p-Mst1, p-YAP, and YAP expressions in NPMSCs exposed to different concentrations of H_2_O_2_ for 12 h (*n* = 3, *P < 0.05, **P < 0.01, one-way ANOVA followed by Bonferroni’s post hoc test)

CCK-8 experiments showed that 75 μM and 100 μM H_2_O_2_ treatments could significantly improve the proliferation of NPMSCs (OD = 1.76 ± 0.04 *P* < 0.01; OD = 1.37 ± 0.01 *P* < 0.01), whereas 300 μM H_2_O_2_ dramatically inhibited the proliferation ability of NPMSCs (OD = 0.20 ± 0.05 *P* < 0.01) (Fig. 2a). The results of EdU staining showed that compared to the 0 μM H_2_O_2_ group (12.2% ± 1.8%), after 75 μM H_2_O_2_ treatment, the percentage of EdU-positive cells increased to 33. 0% ± 1.9% (*P* < 0.01), while 300 μM H_2_O_2_ reduced the percentage of EdU-positive cells to 5.7% ± 1.2% (*P* < 0.01) (Fig. 2b, c). Western blot results showed that 75 μM H_2_O_2_ treatment elevated the expression of cyclin D1 and suppressed the expression of cyclin-dependent kinase inhibitor 2A (P16) (*P* < 0.01). In comparison, 300 μM H_2_O_2_ treatment significantly inhibited the expression of cyclin D1 and significantly increased the expression of P16 (*P* < 0.01) (Fig. 2d, e).

Western blotting was used to detect the expression of key proteins in the Hippo pathway. The results showed that 75 μM H_2_O_2_ treatment reduced the phosphorylation levels of key proteins in the Hippo pathway including phosphorylated large tumour suppressor kinase 1 (p-Mst 1) (*P* < 0.01), phosphorylated mercaptopyruvate sulfurtransferase-1 (p-Lats1) (*P* < 0.05), and phosphorylated Yes1-associated transcriptional regulator (p-YAP) (*P* < 0.01) (Fig. 2f, g). However, NPMSCs that received 300 μM H_2_O_2_ treatment exhibited the opposite result.

### Pretreatment with 75 μM H_2_O_2_ enhanced the antioxidative stress capacity of NPMSCs

**Fig. 3:**
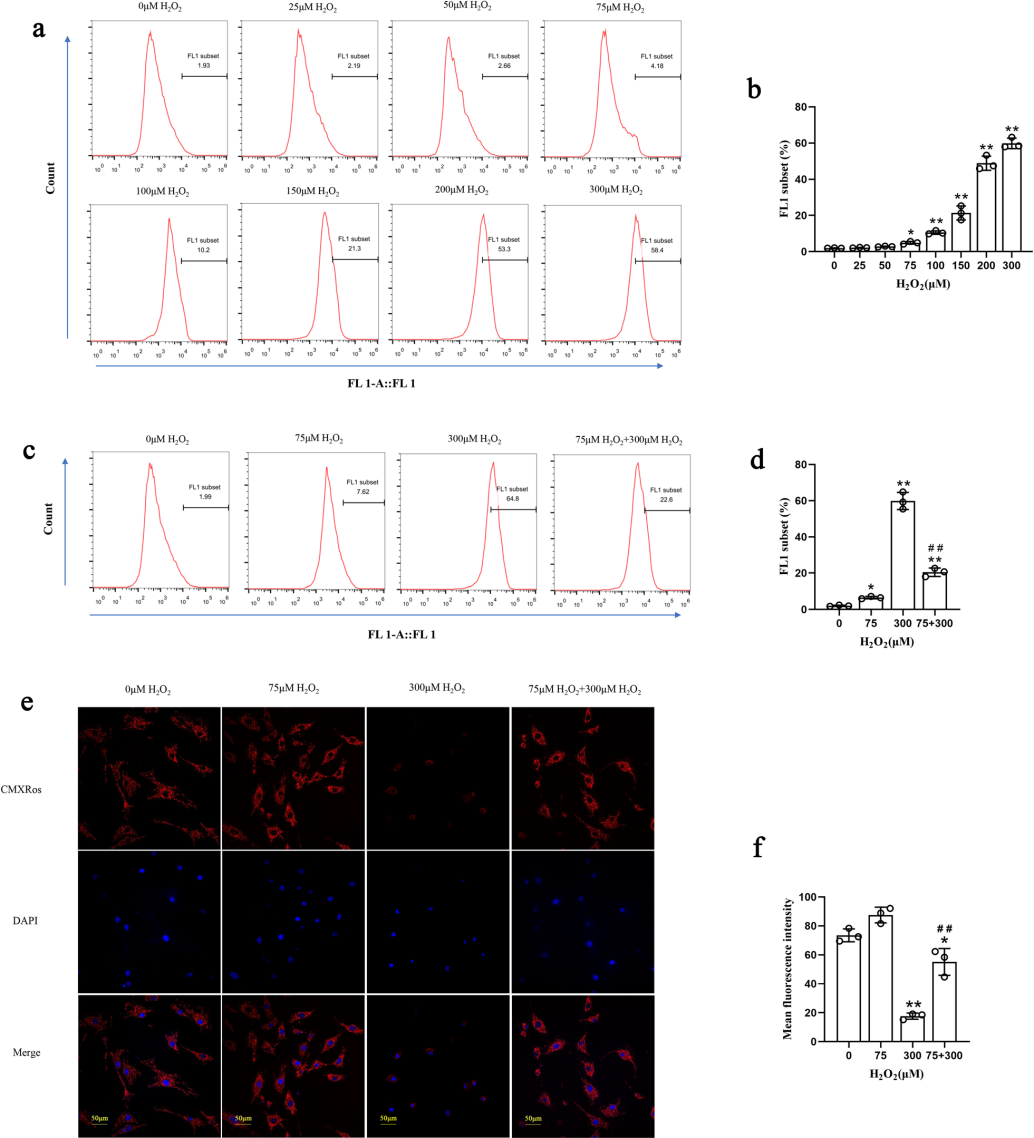
Effect of different H_2_O_2_ concentrations on ROS and MMP in NPMSCs. **a** and **b** The intracellular ROS in NPMSCs that received different H_2_O_2_ concentrations were detected by flow cytometry and quantified (*n* = 3, *P < 0.05, **P < 0.01, one-way ANOVA followed by Bonferroni’s post hoc test). **c** and **d** The intracellular ROS in NPMSCs that received 75 μM H_2_O_2_ pretreatment were detected by flow cytometry and quantified (*n* = 3, *P < 0.05, **P < 0.01, one-way ANOVA followed by Bonferroni’s post hoc test). **e** and **f** The MMP of NPMSCs received 75 μM H_2_O_2_ pretreatment detected by CMXRos and quantified (*n* = 3, *P < 0.05, **P < 0.01, one-way ANOVA followed by Bonferroni’s post hoc test, ^##^*P* < 0.01 compared to the 300 μM H_2_O_2_ group). Scale bar = 50 μm

The intracellular ROS content was detected under different concentrations of H_2_O_2_ treatment by flow cytometry. In the pretreatment process, we found that with increasing H_2_O_2_ concentration, the intracellular ROS concentration also increased (Fig. 3a, b). However, compared with the 300 μM H_2_O_2_ group (59.8% ± 4.4%), NPMSCs that received 75 μM H_2_O_2_ pretreatment for 12 h showed significantly lower ROS content when they were exposed to 300 μM H_2_O_2_ again (20.4% ± 2.5% *P* < 0.01) (Fig. 3c, d).

The MitoTracker Red CMXRos probe was used to detect the MMP. The results showed that the mean fluorescence intensity of CMXRos in NPMSCs treated with 300 μM H_2_O_2_ was significantly reduced (17.6 ± 2.4 *P* < 0.01). However, after pretreatment with 75 μM H_2_O_2_, the mean fluorescence intensity of cells was higher than the mean fluorescence intensity of cells treated with 300 μM H_2_O_2_ alone (55.1 ± 10.4 *P* < 0.01) (Fig. 3e, f).

### Pretreatment with 75 μM H_2_O_2_ improved the resistance of NPMSCs to oxidative stress by inhibiting Brd4 and activating the Keap1-Nrf2 signalling pathway

**Fig. 4:**
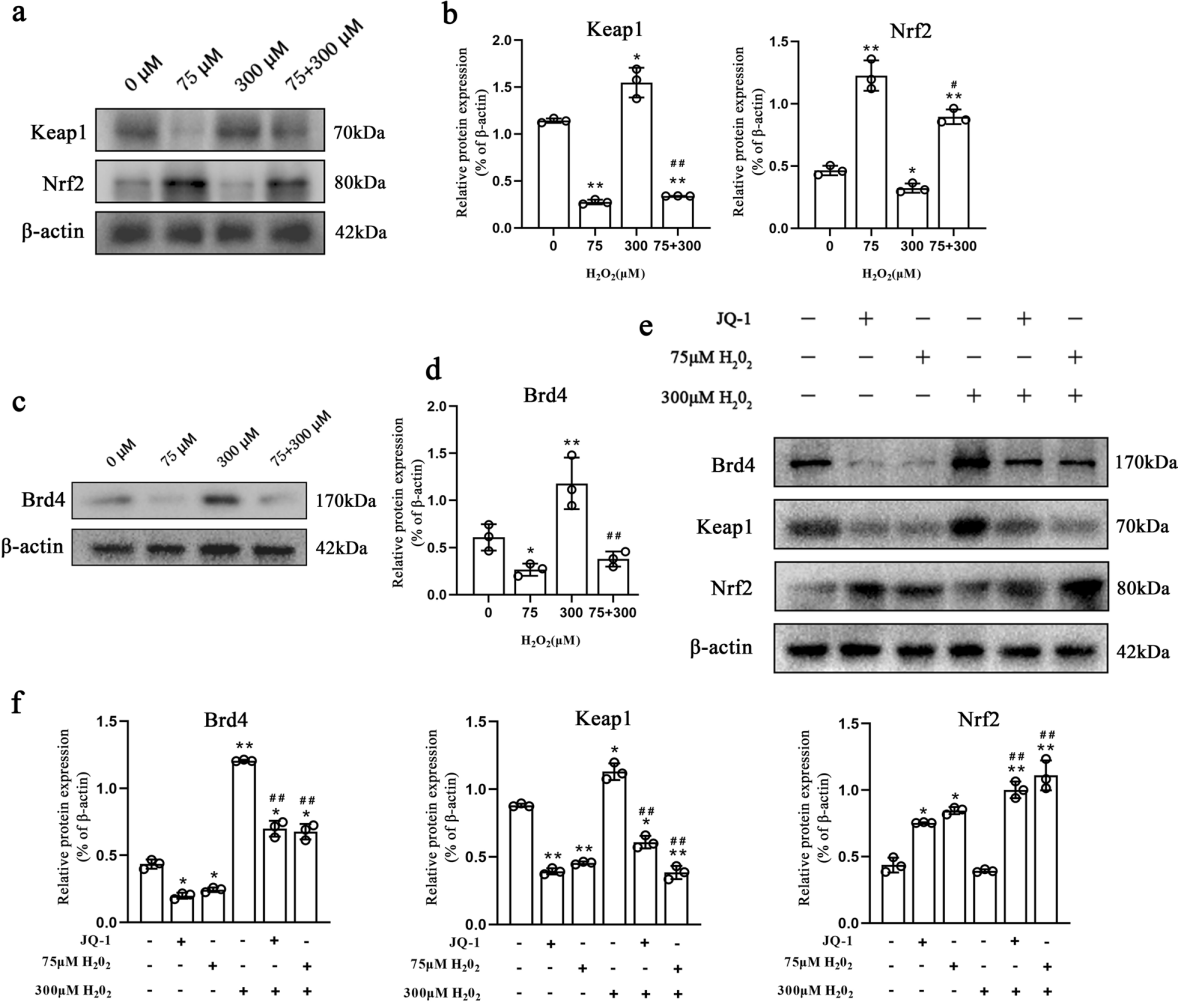
Effect of different H_2_O_2_ concentrations on the Keap1-Nrf2 signalling pathway in NPMSCs. **a** and **b** Western blotting analysis of Keap1 and Nrf2 expression in NPMSCs exposed to different concentrations of H_2_O_2_ (*n* = 3, *P < 0.05, **P < 0.01, one-way ANOVA followed by Bonferroni’s post hoc test. ^#^*P* < 0.05 compared to the 300 μM H_2_O_2_ group). **c** and **d** Western blotting analysis of Brd4 expression in NPMSCs exposed to different concentrations of H_2_O_2_ (*n* = 3, *P < 0.05, **P < 0.01, one-way ANOVA followed by Bonferroni’s post hoc test. ^#^*P* < 0.01 compared to the 300 μM H_2_O_2_ group). **e** and **f** Western blotting analysis estimating the effect of 75 μM H_2_O_2_ pretreatment on Keap1, Nrf2, and Brd4 expression and quantification when NPMSCs were subsequently exposed to 300 μM H_2_O_2_ (*n* = 3, *P < 0.05, **P < 0.01, one-way ANOVA followed by Bonferroni’s post hoc test. ^##^*P* < 0.01 compared to the 300 μM H_2_O_2_ group)

We detected the expression of key proteins in the Keap1-Nrf2 signalling pathway. Western blotting results showed that when NPMSCs pretreated with 75 μM H_2_O_2_ were exposed to 300 μM H_2_O_2_, the expression level of Keap1 declined significantly (*P* < 0.01), and the expression level of Nrf2 dramatically rose compared to NPMSCs exposed to 300 μM H_2_O_2_ alone (*P* < 0.01) (Fig. 4a, b).

We found that compared with exposure to 300 μM H_2_O_2_ alone, Brd4 expression in NPMSCs pretreated with 75 μM H_2_O_2_ decreased significantly when the NPMSCs were exposed to 300 μM H_2_O_2_ (*P* < 0.01) (Fig. 4c, d). Furthermore, we treated 75 μM H_2_O_2_-pretreated NPMSCs with the Brd4 inhibitor JQ-1 (13030, MCE, Shanghai, China) and then exposed the cells to 300 μM H_2_O_2_. Western blotting results showed that Brd4 expression in cells in the JQ-1 treatment group and 75 μM H_2_O_2_ pretreatment group decreased tremendously (*P* < 0.01). Meanwhile, the expression level of Keap1 was significantly decreased and the expression level of Nrf 2 was significantly elevated (*P* < 0.01) (Fig. 4e, f).

### Pretreatment with 75 μM H_2_O_2_ enhanced the antiapoptotic capacity of NPMSCs

**Fig. 5:**
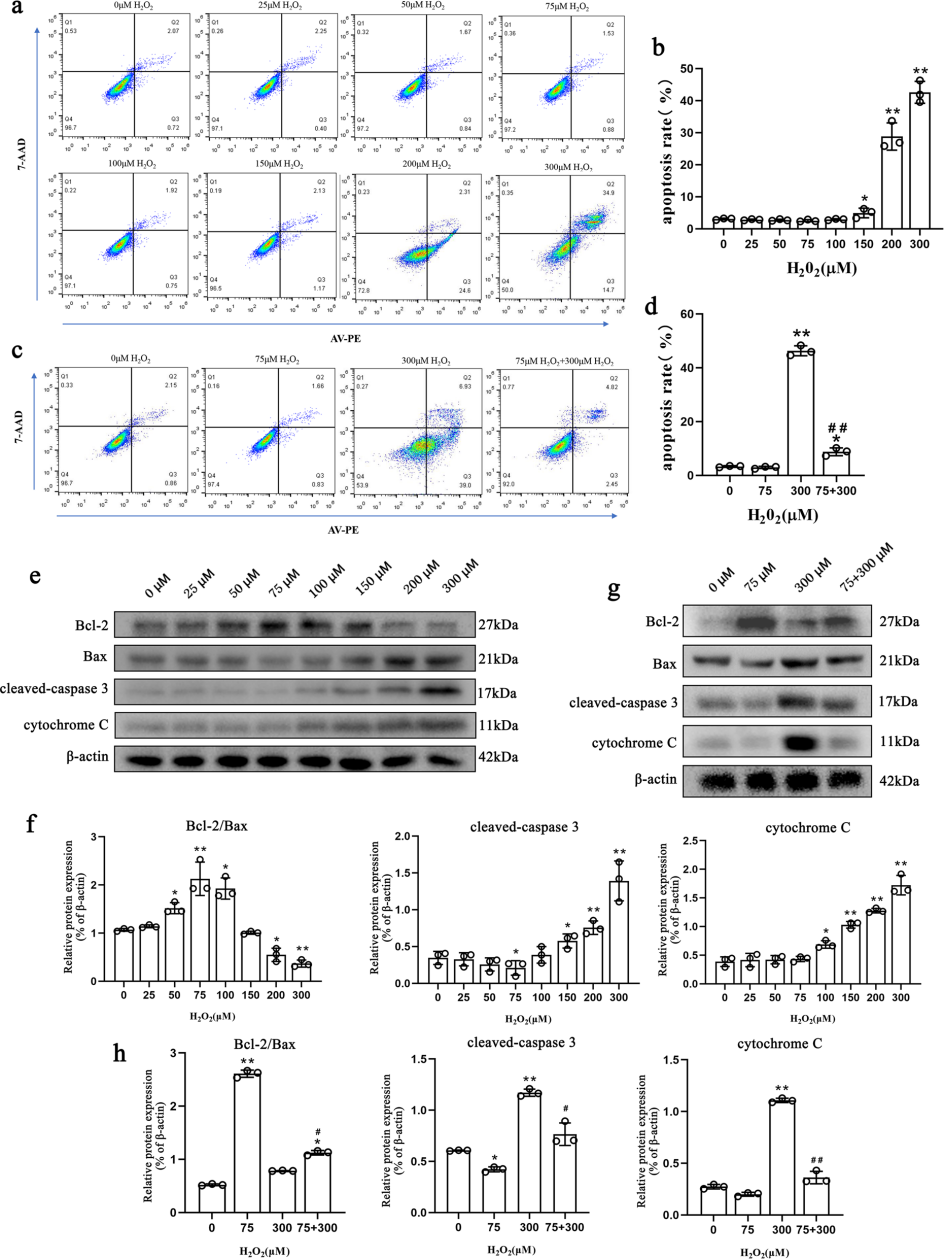
Effect of different H_2_O_2_ concentrations on the antiapoptotic capacity of NPMSCs. **a** and **b** Effect of different H_2_O_2_ concentrations on the apoptosis ratio detected by flow cytometry and quantification (*n* = 3, *P < 0.05, **P < 0.01, one-way ANOVA followed by Bonferroni’s post hoc test). **c** and **d** Effect of 75 μM H_2_O_2_ pretreatment on the apoptosis ratio detected by flow cytometry and quantification when NPMSCs were subsequently exposed to 300 μM H_2_O_2_ (*n* = 3, *P < 0.05, **P < 0.01, one-way ANOVA followed by Bonferroni’s post hoc test. ^##^*P* < 0.01 compared to the 300 μM H_2_O_2_ group). **e** and **f** Western blotting analysis estimating the effect of different H_2_O_2_ concentrations on Bcl-2, Bax, cleaved caspase-3, and cytochrome C expression and quantification (*n* = 3, *P < 0.05, **P < 0.01, one-way ANOVA followed by Bonferroni’s post hoc test). **g** and **h** Western blotting analysis estimating the effect of 75 μM H_2_O_2_ pretreatment on Bcl-2, Bax, cleaved caspase-3, and cytochrome C expression and quantification when NPMSCs were subsequently exposed to 300 μM H_2_O_2_ (*n* = 3, *P < 0.05, **P < 0.01, one-way ANOVA followed by Bonferroni’s post hoc test. ^#^*P* < 0.05, ^##^*P* < 0.01 compared to the 300 μM H_2_O_2_ group)

The results of flow cytometry showed that when the treatment concentration of H_2_O_2_ was less than 150 μM, there was no statistically significant change in the percentage of apoptotic NPMSCs. The percentage of apoptosis increased when NPMSCs were treated with 200 μM (29.8% ± 3.9%, *P* < 0.01) and 300 μM (44.1% ± 2.2%, *P* < 0.01) H_2_O_2_ (Fig. 5a, b). However, when NPMSCs were pretreated with 75 μM H_2_O_2_ for 12 h, the percentage of apoptotic cells was significantly reduced (8.71% ± 1.44% *P* < 0.01) when they were subsequently exposed to 300 μM H_2_O_2_ (Fig. 5c, d).

We examined the effects of different concentrations of H_2_O_2_ on mitochondrial apoptosis-related proteins. The results showed that 75 μM H_2_O_2_ treatment exhibited the highest ratio of Bcl-2 to Bax (*P* < 0.01). Over 100 μM H_2_O_2_ led to distinctly increased cytochrome C levels. Increased cleaved caspase-3 levels were observed when the concentrations of H_2_O_2_ exceeded 150 μM (Fig. 5e, f). However, compared with NPMSCs treated with 300 μM H_2_O_2_-treated NPMSCs, NPMSCs pretreated with 75 μM H_2_O_2_ demonstrated an elevated Bcl-2/Bax ratio (*P* < 0.05), decreased cleaved caspase-3 (*P* < 0.05) and suppressed cytochrome C (*P* < 0.01) when they were subsequently exposed to 300 μM H_2_O_2_ (Fig. 5g, h). The overall in vitro experimental flow chart and timetable are shown in Additional file 1: Fig. S1.

### Pretreatment with 75 μM H_2_O_2_ promoted the therapeutic effects of transplanted NPMSCs in treating IVDD

**Fig. 6:**
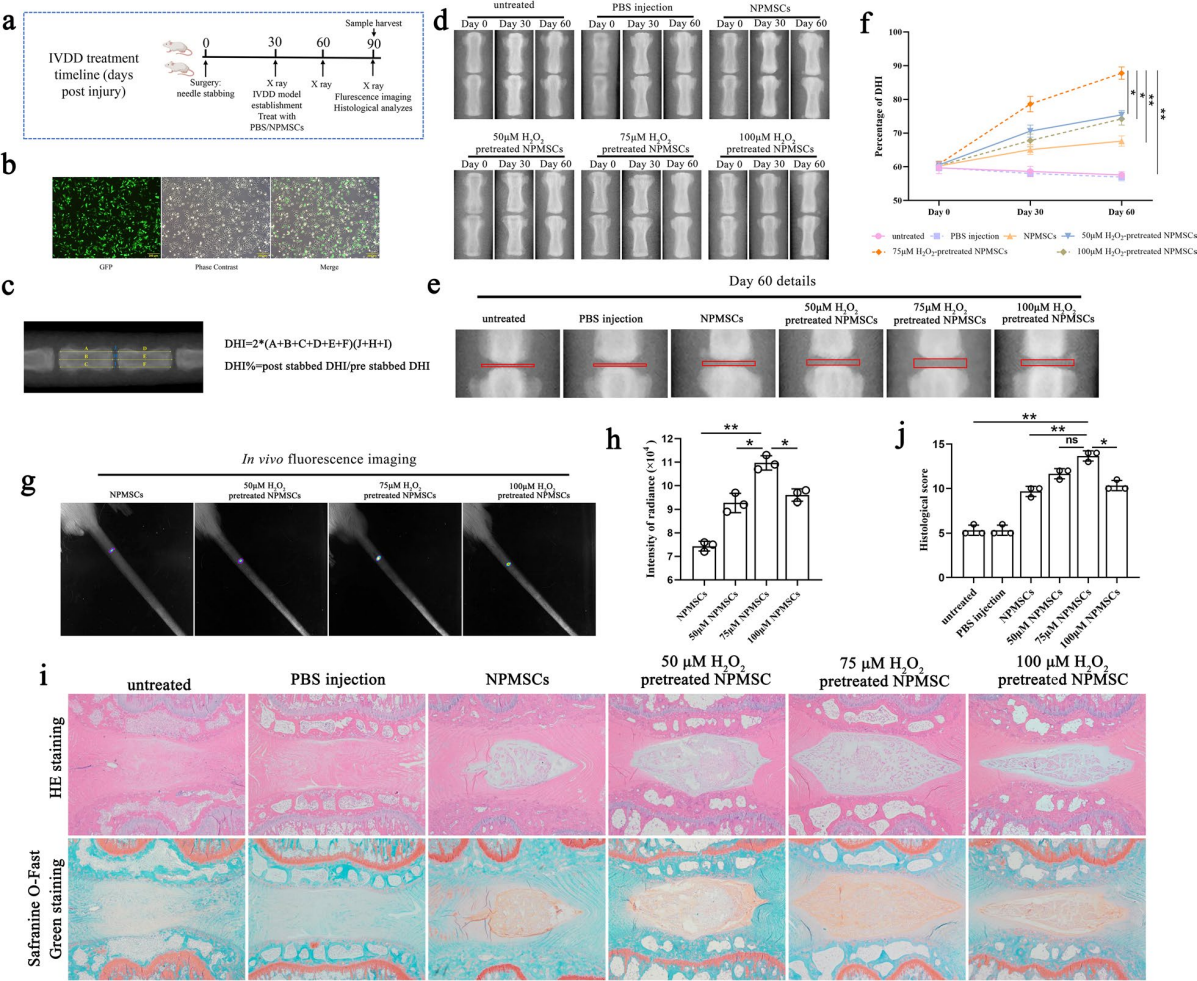
Pretreatment with 75 μM H_2_O_2_ facilitated the therapeutic effects of transplanted NPMSCs in treating IVDD. **a** Flow chart and timetable of rat treatment from day 0 to day 90. **b** GFP was overexpressed in NPMSCs utilizing lentiviral vectors to trace the transplanted cells. Scale bar = 200 μm. **b** Representative X-ray images were taken on the 0, 30th, and 60th days after injection. **c** Methods of calculating DHI%. **d**–**f** Quantification of DHI% in rats on designated days after treatment. (*n* = 6, two-way repeated-measures ANOVA followed by Bonferroni’s post hoc test. *P < 0.05, **P < 0.01). **g** and **h** On the 60th day after treatment, the fluorescence intensity of GFP-labelled NPMSCs in intervertebral discs was detected by the multimode small animal live imaging system (*n* = 3, one-way ANOVA followed by Bonferroni’s post hoc test). **i** Representative HE staining and Safranine O-Fast Green staining illustrating the histological structure of the NP tissue at 60 days after treatment. Scale bar = 500 μm. **j** Histological score of the intervertebral disc in each group (*n* = 3, one-way ANOVA followed by Bonferroni’s post hoc test. *P* < 0.05, **P < 0.01, *ns* no statistical difference)

The overall in vivo experimental flow chart and timetable are shown in Fig. 6a. First, we established rat models of IVDD. Then, the GFP was successfully overexpressed in NPMSCs to trace the transplanted cells (Fig. 6b). Next, NPMSCs pretreated with 50 μM, 75 μM, or 100 μM H_2_O_2_ were injected into degenerative intervertebral discs, and X-rays were taken on the 30th and 60th days after transplantation (Fig. 6d, e). The DHI% of rat caudal intervertebral disc was calculated according to a previously reported calculation method [10] (Fig. 6c). On the 60th day after transplantation, we found that all groups with cell transplantation showed an increase in DHI% compared to the untreated group (DHI% = 49.00 ± 1.52) and PBS injection group (DHI% = 46.57 ± 4.00). The treatment effect of the 75 μM H_2_O_2_-pretreated NPMSCs group (DHI% = 90.00 ± 4.55 *P* < 0.01) was better than the treatment effect of the NPMSCs group (DHI% = 69.51 ± 3.31), 50 μM H_2_O_2_-pretreated NPMSCs group (DHI% = 74.85 ± 1.89) and 100 μM H_2_O_2_-pretreated NPMSCs group (DHI% = 74.12 ± 2.16) (Fig. 6d–f).

In vivo fluorescence imaging results showed that obvious fluorescence could be observed in the caudal intervertebral disc of all groups of rats transplanted with NPMSCs. The fluorescence intensity of the rats injected with NPMSCs pretreated with 75 μM H_2_O_2_ was higher than the fluorescence intensity of the 50 μM H_2_O_2_-pretreated NPMSCs group and 100 μM H_2_O_2_-pretreated NPMSCs group (*P* < 0.05) and NPMSCs group (*P* < 0.01) (Fig. 6g, h).

The histological structure of the NP tissue on the 60th day after transplantation was analysed by HE staining, Safranine O-Fast Green staining, and histological scores of IVDD (Fig. 6i, j). Reduced disc contents and disordered fibrous tissue were observed in the untreated group and the PBS group. Compared to the untreated group, the histological scores of the four cell transplantation groups were higher, while compared with the NPMSCs group (9.67 ± 0.33), the 75 μM H_2_O_2_-pretreated NPMSCs group exhibited the optimal ameliorative outcome (10.33 ± 0.33 *P* < 0.01).

## Discussion

Although many studies have reported that stem cell transplantation is a feasible method in the treatment of IVDD, the insufficient efficiency of transplantation and low survival rate of grafts limit the therapeutic potential of this method to some extent [13, 31]. A high concentration of H_2_O_2_ can induce a surge in intracellular ROS, which damages organelles and results in cell cycle arrest and cell apoptosis [27]. However, pretreatment of transplanted cells with H_2_O_2_ at appropriate concentrations can result in beneficial effects. For example, pretreatment with H_2_O_2_ enhanced the antiapoptotic ability of BMMSCs in skin wounds [24, 32]. H_2_O_2_ pretreatment of human umbilical cord mesenchymal stem cells (UCMSCs) or human ADMSCs improved the ability of cells to resist high-dose H_2_O_2_-induced oxidative stress [33, 34]. In this study, we explored whether pretreating NPMSCs with H_2_O_2_ can improve the therapeutic efficacy of transplanted NPMSCs in IVDD and identified the optimal concentration. We found that 75 μM H_2_O_2_-pretreated NPMSCs demonstrated the strongest elevated cell proliferation, enhanced antioxidative stress ability, and distinctly reduced apoptosis. The in vivo experimental results verified that 75 μM H_2_O_2_-pretreated NPMSCs transplantation exhibited distinctly enhanced DHI%, better histological morphology, and higher survival. These results demonstrate that 75 μM H_2_O_2_ is an optimal concentration for facilitating the therapeutic potential of NPMSCs transplantation in IVDD treatment.

Stem cells reported to treat IVDD include induced pluripotent stem cells (iPSCs), BMMSCs, ADSCs, and NPMSCs [8, 13, 35]. NPMSCs are stem cells derived from nucleus pulposus tissue that is regarded as a more suitable candidate for transplantation in IVDD treatment. NPMSCs can survive better in the microenvironment of the degenerated intervertebral disc and replenish the residual nucleus pulposus cells [15, 35, 36]. Therefore, NPMSCs were selected as research objects in our study. NPMSCs were isolated from the nucleus pulposus tissue of rats. We observed that these cells could proliferate and grow adherent in a cell culture flask. In addition, these cells expressed CD34 and CD45 at low levels and CD73, CD90, and CD105 at high levels, consistent with the standardized cell surface markers of mesenchymal stem cells [37]. Furthermore, these cells could undergo trilineage differentiation. All of the above characteristics indicated that these cells fit the characteristics of mesenchymal stem cells [37].

To determine the optimal concentration of H_2_O_2_ for NPMSCs pretreatment, NPMSCs were cultured with different concentrations of H_2_O_2_ for 12 h according to the previous article reports [24]. First, the cell viability and cell proliferation of NPMSCs were detected. We found that 50 μM, 75 μM, and 100 μM H_2_O_2_ promoted the viability and proliferation of NPMSCs, while 200 μM H_2_O_2_ and 300 μM H_2_O_2_ inhibited the viability and proliferation of NPMSCs. Among the designated concentrations, 75 μM H_2_O_2_-treated NPMSCs exhibited the highest facilitating effects. Moreover, the expression of cyclin D1, reported as a cell proliferation facilitator [38, 39], as well as P16, a well-known cell cycle repressor [40], was detected to consolidate our results. Consistent with the above observations, we found that 75 μM H_2_O_2_-treated NPMSCs exhibited maximized expression of cyclin D1 and the lowest expression of P16, whereas the expression of cyclin D1 was decreased and the expression of P16 was elevated to the greatest extent in 300 μM H_2_O_2_-treated NPMSCs. Therefore, 75 μM was selected as the pretreatment concentration and 300 μM was selected as the concentration to induce inhibition of cell proliferation in subsequent experiments.

The Hippo pathway plays an important role in regulating stem cell proliferation and apoptosis [27, 41, 42]. Previous studies have shown that the activated Hippo pathway can inhibit cell proliferation and promote cell apoptosis of IVD cells [43–46]. Moreover, the inhibition of the Hippo pathway is closely related to H_2_O_2_-induced BMMSCs proliferation upregulation and apoptosis downregulation [47]. Therefore, the expression of key proteins in the Hippo pathway was detected to explore the mechanism by which H_2_O_2_ promotes NPMSCs proliferation. We found that 75 μM H_2_O_2_ treatment significantly reduced the phosphorylation of Lats1, Mst1, and YAP, indicating that the Hippo pathway was inhibited. However, when NPMSCs were pretreated with 300 μM H_2_O_2_, the phosphorylation of key proteins in this pathway decreased, indicating that 75 μM H_2_O_2_ pretreatment may promote NPMSCs proliferation by inhibiting the Hippo pathway to some extent.

Oxidative stress is one of the key factors leading to cell damage in the intervertebral disc. ROS generated by intracellular metabolism can cause mitochondrial homeostasis loss and induce cell death [48–50]. We found that with increasing H_2_O_2_ concentration, the intracellular ROS content increased gradually. However, we found that 75 μM H_2_O_2_-pretreated NPMSCs exhibited a distinct suppressed ROS level and upregulated MMP when they were supplemented with subsequent 300 μM H_2_O_2_. These outcomes indicate that NPMSCs can activate their own antioxidant system after pretreatment and thus reduce the content of ROS in cells when they encounter detrimental 300 μM H_2_O_2_. The Keap1-Nrf2 signalling pathway is the most important antioxidant system in cells. Activated Nrf2 can promote the expression of antioxidant factors such as NAD(P)H quinone dehydrogenase 1 (NQO1) and superoxide dismutase (SOD) [51–54]. Previous studies have shown that activating the Keap1-Nrf2 signalling pathway could protect IVD cells from oxidative stress injury [55, 56]. Therefore, we then explored whether the decreased ROS and increased MMP were associated with the activation of the Keap1-Nrf2 signalling pathway. Our results found that 75 μM pretreated H_2_O_2_ exhibited enhanced expression of Nrf2, and reduced expression of Keap1 when they were supplemented with subsequent 300 μM H_2_O_2_. As an upstream regulatory molecule of the Keap1-Nrf2 signalling pathway, Brd4 can inhibit the activation of this pathway and elevate intracellular oxidative stress [57, 58]. Interestingly, when NPMSCs were treated with 75 μM H_2_O_2_, Brd4 was also decreased. Therefore, we hypothesized that pretreatment with 75 μM H_2_O_2_ might regulate the Keap1-Nrf2 signalling pathway by regulating Brd4. Subsequently, we treated NPMSCs with the Brd4 inhibitor JQ-1. JQ-1-treated NPMSCs and 75 μM H_2_O_2_-pretreated NPMSCs exhibited similar changes in the expression levels of Brd4, Keap1, and Nrf2, which partially confirmed our hypothesis.

Meanwhile, the apoptotic rate of NPMSCs under different H_2_O_2_ concentrations was detected. We found that 75 μM H_2_O_2_-treated NPMSCs exhibited the lowest cell apoptotic rate, while 300 μM H_2_O_2_-treated NPMSCs exhibited the highest cell apoptotic rate. Moreover, 75 μM H_2_O_2_-pretreated NPMSCs exhibited a distinct suppressed cell apoptotic rate when they were supplemented with subsequent 300 μM H_2_O_2_. The ratio of the antiapoptotic protein Bcl-2 to the proapoptotic protein Bax is an important indicator of the degree of apoptosis mediated by the mitochondrial pathway [52, 59]. The level of cytochrome C is the gold standard for mitochondrial damage [60]. Therefore, the expression levels of Bcl-2, Bax, apoptotic marker cleaved caspase-3, and cytochrome C were detected. We found that 75 μM H_2_O_2_-treated NPMSCs demonstrated the highest ratio of Bcl-2 to Bax, and the lowest levels of cleaved caspase-3 and cytochrome C levels. The NPMSCs that received 300 μM H_2_O_2_ demonstrated the lowest ratio of Bcl-2 to Bax and the highest levels of cleaved caspase-3 and cytochrome C. However, NPMSCs supplemented with 300 μM H_2_O_2_ exhibited an elevated ratio of Bcl-2 to Bax and reduced levels of cleaved caspase-3 and cytochrome C levels if they were pretreated with 75 μM H_2_O_2_. These results suggest that pretreatment of NPMSCs with 75 μM H_2_O_2_ may enhance the ability of cells to resist apoptosis by stabilizing mitochondria. These beneficial effects may also partly be attributed to the effects of H_2_O_2_ on the Hippo pathway and Keap1-Nrf2 pathway.

**Fig. 7:**
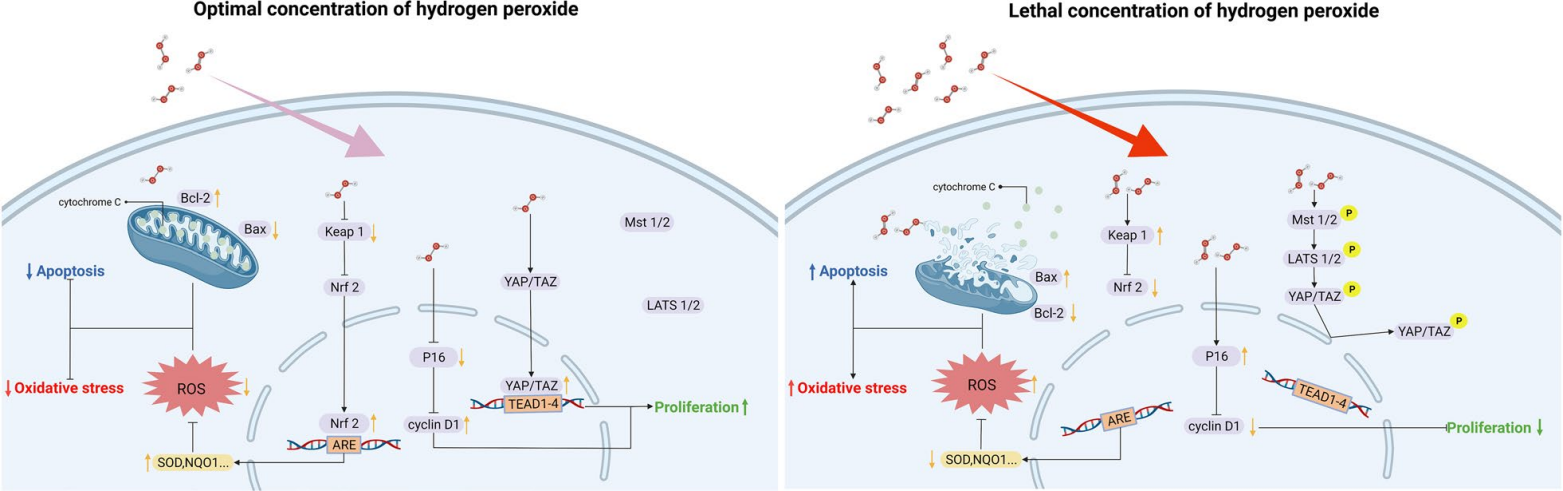
Signalling pathway involved in H_2_O_2_ regulation of cell proliferation, oxidative stress, and cell apoptosis in NPMSCs. The optimal concentration of H_2_O_2_ was 75 μM; the lethal concentration of H_2_O_2_ was 300 μM. Up arrow: upregulation. Down arrow: downregulation

The above experimental results indicate that pretreatment with 75 μM H_2_O_2_ for 12 h can significantly improve the antiapoptotic ability of NPMSCs. Further research will illuminate the underlying mechanisms by which H_2_O_2_ pretreatment regulates NPMSCs apoptosis. The signalling pathways involved in the regulation of cell proliferation, oxidative stress, and cell apoptosis by H_2_O_2_ are summarized in Fig. 7.

Based on the above in vitro study, we determined that 75 μM H_2_O_2_ pretreatment may maximize the proliferation and antiapoptotic ability of NPMSCs. Therefore, we further explored whether 75 μM H_2_O_2_ pretreatment NPMSCs could improve the therapeutic efficacy of NPMSCs transplantation in a rat IVDD model. As expected, compared to rats in other groups, rats injected with 75 μM H_2_O_2_-pretreated NPMSCs showed increased transplanted cell survival, facilitated extracellular matrix deposition, and a distinct improvement in DHI% during a 60-day observation. Therefore, these results confirmed that 75 μM H_2_O_2_ is an optimal concentration for NPMSCs’ pretreatment before transplantation. Notably, the optimal concentration of H_2_O_2_ pretreatment was slightly higher than the optimal concentration of previous studies [61, 62]. This discrepancy may be attributed to the intrinsic features of NPMSCs, which reside in a microenvironment of high mechanical pressure, low oxygen supplementation, and high lactic acid concentration. NPMSCs may innately possess a certain resistance to H_2_O_2_.

There are several limitations in our experiment. First, our experiment directly selected the time of H_2_O_2_ pretreatment reported before; thus, the relationship between the therapeutic effects of H_2_O_2_ and pretreatment time was not explored. A time gradient experiment will further explore the optimal invention time to maximize the facilitating effects of H_2_O_2_ in the future. In addition, the underlying mechanisms by which H_2_O_2_ pretreatment regulates the Hippo signalling pathway and Keap1-Nrf2 signalling pathway were not investigated in our current experiments, and further molecular-level understanding of the experimental observations needs to be explored in the future. However, the present experiments have proven the beneficial effects of 75 μM H_2_O_2_ in NPMSCs pretreatment. Given that this method is convenient and efficient, H_2_O_2_-pretreated NPMSCs transplantation may become a feasible method for treating IVDD clinically in the future.

## Conclusions

In summary, we identified for the first time that pretreatment with 75 μM H_2_O_2_ can better promote cell proliferation and reduce oxidative stress and cell apoptosis in NPMSCs in vitro, which is related to the regulation of the Hippo signalling pathway and Keap1-Nrf2 pathway. NPMSCs pretreated with 75 μM H_2_O_2_ also exhibited the most satisfactory therapeutic effects when transplanted into rat degenerated caudal intervertebral discs. These results demonstrate that 75 μM H_2_O_2_ is an optimal concentration for NPMSCs pretreatment and may serve as a feasible method for the clinical application of NPMSCs-based therapy in treating IVDD in the future.

## Supplementary Information

**Additional file 1: Fig. S1.** Flow chart and timetable of rat NPMSCs treatment process. Group A was pretreatment group; Group B was unpretreatment group.

**Additional file 2: Table S1**. Histological grading scale of the disc degeneration.

## Data Availability

All the data supporting the results were shown in the article and can be applicable from the corresponding author on reasonable request.

## Abbreviations

IVDD: Intervertebral disc degeneration
NPMSCs: Nucleus pulposus mesenchymal stem cells
H_2_O_2_: Hydrogen peroxide
ROS: Reactive oxygen species
MMP: Mitochondrial membrane potential
Brd4: Bromodomain-containing protein 4
Keap1: Kelch-like ECH-associated protein 1
Nrf2: Nuclear factor, erythroid-derived 2, like 2
BMMSCs: Bone marrow mesenchymal stem cells
OD: Optical density
GFP: Green fluorescent protein
MOI: Multiplicity of infection
CO6-7: Coccyx 6–7
HE: Haematoxylin–eosin
UCMSCs: Umbilical cord mesenchymal stem cells
ADMSCs: Adipose mesenchymal stem cells
iPSCs: Induced pluripotent stem cells
P16: Cyclin-dependent kinase inhibitor 2A
p-Lats1: Phosphorylated large tumour suppressor kinase 1
p-Mst1: Phosphorylated mercaptopyruvate sulfurtransferase-1
p-YAP: Phosphorylated Yes1-associated transcriptional regulator
YAP: Yes1-associated transcriptional regulator
TAZ: Tafazzin
ARE: Antioxidant response elements
NQO1: NAD(P)H quinone dehydrogenase 1
SOD: Superoxide dismutase
