# Supplementary figures and images for: Pretreatment of nucleus pulposus mesenchymal stem cells with appropriate concentration of H2O2 enhances their ability to treat intervertebral disc degeneration
Source: Stem Cell Res Ther. 2022 Jul 26;13:340. doi: 10.1186/s13287-022-03031-7 (PMC9327256; doi:10.1186/s13287-022-03031-7)

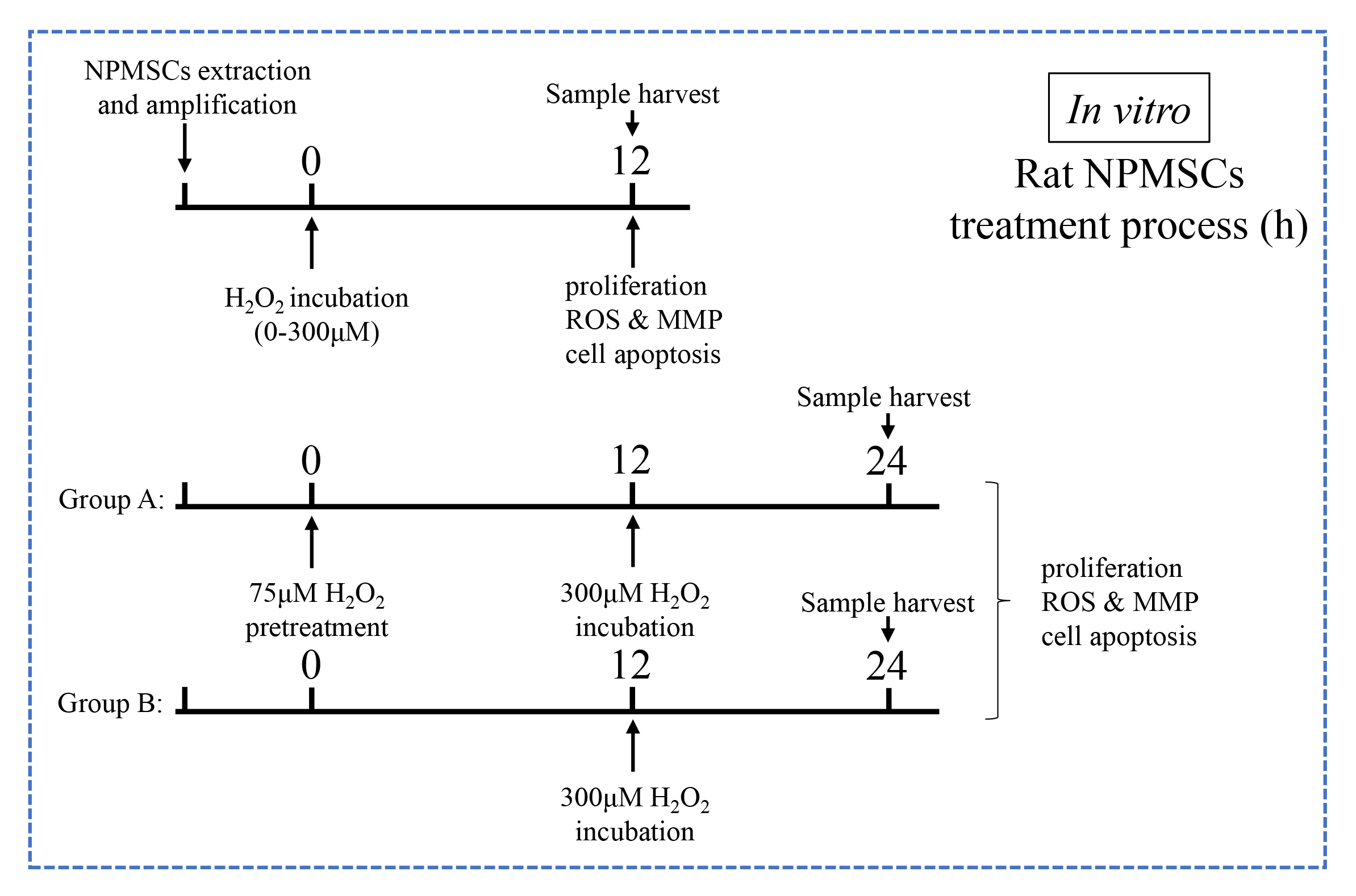

Supplement: Supplementary file 1 — Additional file 1: Fig. S1. Flow chart and timetable of rat NPMSCs treatment process. Group A was pretreatment group; Group B was unpretreatment group. [file 13287_2022_3031_MOESM1_ESM.jpg]
