# Supplementary material for: Pretreatment of nucleus pulposus mesenchymal stem cells with appropriate concentration of H2O2 enhances their ability to treat intervertebral disc degeneration
Source: Stem Cell Res Ther. 2022 Jul 26;13:340. doi: 10.1186/s13287-022-03031-7 (PMC9327256; doi:10.1186/s13287-022-03031-7)
Supplement: Supplementary file 2 — Additional file 2: Table S1. Histological grading scale of the disc degeneration. [file 13287_2022_3031_MOESM2_ESM.docx]

| Histological grading of the disc degeneration seen from inner annulus structure |
| --- |
| *I. Cellularity of the anulus ﬁbrosus*  Grade:  1. Fibroblasts comprise more than 75% of the cells  2. Neither ﬁbroblasts nor chondrocytes comprise more than 75% of the cells  3. Chondrocytes comprise more than 75% of the cells  *II. Morphology of the anulus ﬁbrosus*  Grade:  1. Well-organized collagen lamellae without ruptured or serpentine ﬁbers  2. Inward bulging, ruptured or serpentine ﬁbers in less than one third of the annulus  3. Inward bulging, ruptured or serpentine ﬁbers in more than one third of the annulus  *III. Border between the anulus ﬁbrosus and nucleus pulposus*  Grade:  1. Normal, without any interruption  2. Minimal interruption  3. Moderate or severe interruption  *IV. Cellularity of the nucleus pulposus*  Grade:  1. Normal cellularity with stellar shaped nuclear cells evenly distributed throughout the nucleus  2. Slight decrease in the number of cells with some clustering  3. Moderate or severe decrease (>50%) in the number of cells with all the remaining cells clustered and separated by dense areas of proteoglycans  *V. Morphology of the nucleus pulposus*  Grade:  1. Round, comprising at least half of the disc area in midsagittal sections  2. Rounded or irregularly shaped, comprising one quarter to half of the disc area in midsagittal sections  3. Irregularly shaped, comprising less than one quarter of the disc area in midsagittal sections |
